# Successful Repair and Management of Severe Scalp Avulsion Incurred during Birth in an Extremely Low Birth Weight Infant

**DOI:** 10.1155/2024/8122801

**Published:** 2024-09-10

**Authors:** Shoko Takahashi, Yu Kanai, Yayoi Miyazono, Daisuke Hitaka, Yuki Fujita, Yoichiro Shibuya, Hidetoshi Takada

**Affiliations:** ^1^ Department of Pediatrics University of Tsukuba Hospital, Tsukuba, Japan; ^2^ Department of Child Health Institute of Medicine University of Tsukuba, Tsukuba, Japan; ^3^ Department of Plastic and Reconstructive Surgery Institute of Medicine University of Tsukuba, Tsukuba, Japan

## Abstract

**Introduction:**

Minor head trauma, such as scalp abrasion, is relatively common during vaginal delivery, whereas fatal head trauma is rare. This case report describes the successful repair and management of severe scalp avulsion that occurred during vaginal delivery and consequent hemorrhagic shock in an extremely low birth weight infant. *Case Presentation*. An extremely low birth weight infant (26 weeks' gestational age) sustained extensive scalp avulsion during vaginal delivery that exposed the skull. The scalp laceration began in the frontal temporal region and extended bilaterally along the temporal region for 20 cm. The infant experienced hemorrhagic shock soon after birth due to bleeding from the wound and was placed in a closed incubator for intensive care. At 7 h after birth, the wounds were repaired using sutures. Bleeding was quickly controlled, and the infant recovered from hemorrhagic shock. A wet dressing was applied to the wound, and the flap healed without necrosis.

**Conclusion:**

We successfully repaired severe scalp avulsion in this case. Scalp avulsion can cause severe bleeding and death. Bleeding control and the preservation of circulation are the most important factors in its repair and maintenance. In this case, suturing the wound effectively controlled the bleeding, and the application of wet dressing and a high-humidity environment thereafter may have contributed to the scalp's engraftment.

## 1. Introduction

Physical forces encountered during parturition can induce injuries. Head injuries, clavicular fractures, and peripheral nerve injuries are associated with vaginal delivery. Head injuries, such as scalp abrasions, caput succedaneum, and cephalohematoma, are the most common, usually minor, and usually require no treatment [1, 2]. However, serious head injuries can occur. Subgaleal hemorrhages, depressed skull fractures, and intracranial hemorrhages are relatively common and require urgent interventions [3]. We report a rare case of severe scalp avulsion that occurred during vaginal delivery. Scalp avulsion typically causes massive bleeding, which can be fatal. Here, we report the successful repair and management of a case of severe scalp avulsion in an extremely low birth weight infant.

## 2. Case Presentation

The female infant was the first dichorionic diamniotic twin born to a 28-year-old mother with no significant medical history (gravida 2, para 1) who conceived using an ovulation-inducing agent. At 26 weeks' gestation, the mother complained of painful uterine contractions and membrane rupture. An emergency cesarean section was performed because of the breech presentation of the second baby. However, when initiated, the head of the first infant was visible from the cervix. The obstetrician attempted to return the first baby to the uterus and deliver it transabdominally. Ultimately, the first baby was delivered vaginally, whereas the second baby was delivered via cesarean section.

The first infant was female with Apgar scores of 1/10 and 5/10 at 1 and 5 min, respectively. An almost complete scalp avulsion involving only the connection to the nuchal area revealed the infant's skull. Furthermore, no spontaneous breathing was observed, and the infant's heart rate was less than 100 beats/min. Positive pressure ventilation was started immediately with a bag and mask, and the infant was intubated 5 min after birth. Thereafter, her heart rate promptly increased. Upon arrival at the neonatal intensive care unit, she was placed on a mechanical ventilator in a closed incubator at an appropriate temperature and high humidity to maintain her body temperature because of her extremely low birth weight.

**Figure 1:**
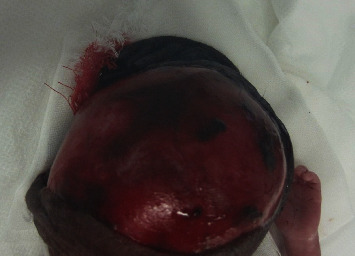
Avulsed bluntly lacerated scalp with the exposed skull noted shortly after birth.

**Figure 2:**
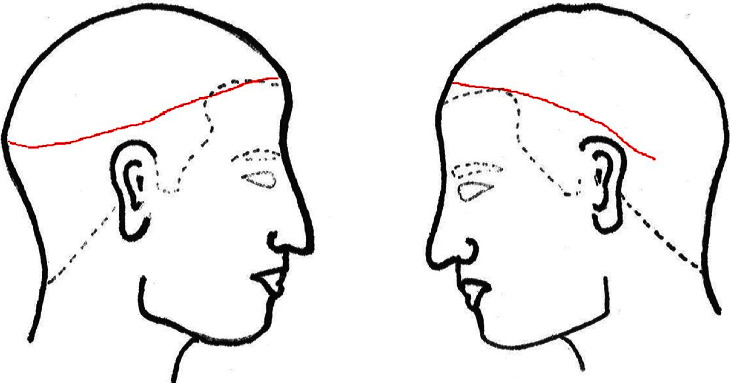
Schematic representation of the laceration (red line). The wound was circumscribed around the left occipital area.

The infant's birth weight, height, and head circumference were 847 g (standard deviation (SD), −0.41), 31.8 cm (SD, −1.21), and 26 cm (SD, +1.38), respectively. Physical examination revealed a blunt laceration of the scalp that started at the frontal temporal region and extended 20 cm along the bilateral temporal regions. The frontoparietal flap was turned over and attached to the left occipital region (Figures 1 and 2). No other physical abnormalities were observed.

**Figure 3:**
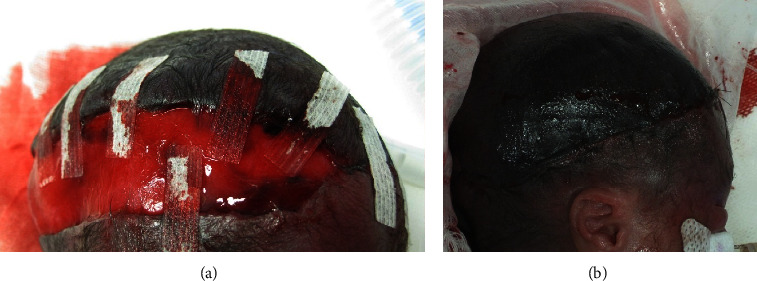
Laceration wound treatment course. (a) The application of adhesive skin-closure strips failed to close the wound and bleeding continued. (b) Mattress sutures were applied, which led to hemostasis.

**Figure 4:**
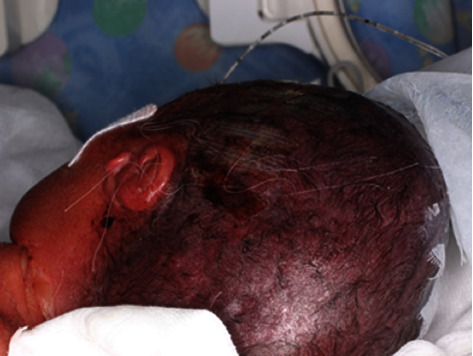
At 72 h after birth, the flap discoloration was relieved and the skin was not necrotic.

**Figure 5:**
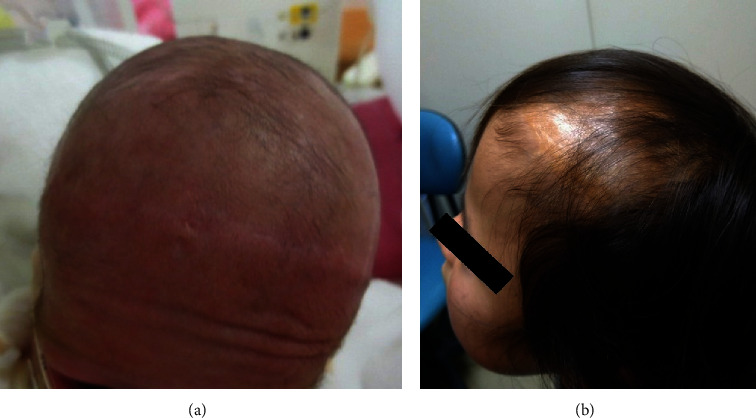
(a) Hair growth from the flap at 5 months after birth. (b) Less hair growth noted on the skin near the transected edge of the flap at 6 years after birth.

Considering that the scalp was thin and that pulling on it would cause immediate flap necrosis, the laceration was first treated using adhesive skin-closure strips (Figure 3(a)). However, these treatments failed to control the bleeding. Moreover, the infant developed hemorrhagic shock 6 hours after birth because of persistent bleeding from the wound. Plastic surgeons applied 5-0 nylon mattress sutures 7 hours after birth, which briefly suppressed bleeding (Figure 3(b)). Owing to blood loss, the infant's systolic blood pressure dropped to 20 mmHg at one point. Furthermore, her hemoglobin concentration (9.8 g/dL at birth) decreased to 6.1 g/dL. We administered dopamine, dobutamine, and hydrocortisone and transfused red blood cells, platelets, and plasma derivatives at 55, 15, and 45 mL/kg, respectively. Finally, 10 h after birth, the infant recovered from the hemorrhagic shock. After repair, we applied a wet dressing and antibiotic ointment to the wound and covered the scar with a silicone gauze. We avoided applying pressure to the occipital area to maintain the blood flow to the flap. At 72 h after birth, flap discoloration was relieved (Figure 4), indicating that blood flow in the area had improved. Hair eventually grew from the flap (Figure 5).

## 3. Discussion

Herein, we report the case of an extremely low birth weight infant with a severe scalp avulsion. Scalp laceration, not scalp avulsion, is one of the most common injuries that occur during delivery [4]. Notably, most cases were mild and did not require special treatment. To date, only a few cases of severe scalp lacerations have been reported, all of which occurred during emergency cesarean sections due to a direct incision with a surgical knife [5, 6]. Each patient died shortly after delivery due to premature birth, but not scalp laceration; one died of brain laceration and disruption. Hence, there have been no reports on laceration repair. To the best of our knowledge, there are no previous reports of scalp avulsion as a birth injury. However, scalp avulsions are generally more severe than scalp lacerations because the scalp and skull are separated in the former, causing massive bleeding and skin necrosis. Therefore, this is the first report of the successful rescue of an infant with severe scalp injury that occurred during vaginal delivery.

Scalp avulsion, which is typically induced by a high force, occurs most commonly during accidents involving hair entrapment in agricultural or industrial machines [7]. Scalp avulsion occurs upon the application of a strong traction force along the area where the tissues are loosely attached, usually in the supraorbital or neck area, where the muscles have less secure attachments than in other areas [8]. In contrast, in the present case, the avulsion started from the frontal region and extended along the bilateral temporal regions, circling along the largest head circumference. We assumed that the primary cause of the damage was the pushing back of the head after exiting the cervical canal during delivery. During this process, the scalp may have been caught on the wall of the birth canal, probably near the true conjugate, the area between the sacral promontory and symphysis pubis, where the birth canal is the narrowest [9]. The infant's premature development and fragile skin tissue presumably also contributed to the scalp avulsion. Incomplete dilation of the birth canal, potentially due to the prematurity of the birth, might have been a contributing factor. In addition, abnormal fetal rotation could have played a role. However, a definitive assessment of fetal rotation was not feasible due to the emergency cesarean section performed.

The scalp has a rich blood supply provided by the four arteries [10]. Avulsion is a life-threatening injury caused by blood loss, and the rapid cessation of bleeding is crucial [8]. We first attempted to control bleeding using adhesive skin-closure strips. Consequently, we treated the wound with sutures 7 h after birth, and the bleeding promptly stopped. Upon suturing, bleeding was controlled, and circulation gradually improved with the help of blood transfusions and drug administration. We believe that suturing is a crucial turning point in saving an infant's life for the following reasons: the scalp consists of five layers: the skin, subcutaneous tissue, epicranial aponeurosis (galea aponeurosis), loose areolar tissue (subgaleal layer/space), and pericranium. Scalp avulsion generally occurs in the loose areolar tissue, which is the most fragile layer of the five layers [7]. In contrast, the superficial arteries and veins of the scalp run superior to the epicranial aponeurosis through the subcutaneous tissue. Notably, no blood vessels were present at the loose areolar tissue level except for the emissary veins, which connect the superficial and cerebral veins [10]. This suggests that the rupture of the superficial veins at the subcutaneous tissue level is the main source of bleeding in scalp avulsions. We believe that the bleeding stopped in this case because of suturing of the cut wound and application of mild pressure to the laceration stump.

An avulsed or amputated scalp flap can generally tolerate ischemia for 4–6 hours in adults [8]. In this study, although we performed the repair 7 h after birth, the flap was successfully engrafted without necrosis, and hair eventually grew from it. Restoration success can be attributed to two factors. Firstly, we carefully maintained minimal blood flow to the flap. Notably, 10 arteries ran through the scalp (five on each side: supratrochlear, supraorbital, superficial temporal, posterior auricular, and occipital). In our case, all arteries, except the left occipital artery, were damaged by avulsion. As shown in Figure 5, the occipital region of the infant's head had more hair than the frontal region, indicating the importance of blood flow distribution from the occipital artery for flap survival. Secondly, the wet dressing and humid environment prevented the flaps from drying. Following complete separation from the body and subsequent transplantation, a skin flap is usually engrafted after receiving nourishment from the periosteal tissue fluid. The most important aspect of this process is preventing the transplanted tissue from drying. The application of a wet dressing to the flap after suturing and housing the infant in a closed high-humidity incubator may have favored scalp engraftment. In addition, this paper discusses suggestions for addressing hair scarcity in the frontal region at 6 years of age. In the context of scalp avulsion in adults, it has been reported that anastomosis of only one superficial temporal artery out of 10 arteries can result in scalp survival, including hair growth [11]. According to the concept of angiosomes, blood flow can be maintained within two vascular territories from the vascular stem [12]. Therefore, it is recommended to attempt an anastomosis of the superficial temporal artery in similar cases of scalp avulsion. In the present case, the infant was unable to undergo microscopic surgery in the operating room due to her extremely low birth weight and unstable circulation.

## 4. Conclusion

Here, we describe a case of successfully repaired severe scalp avulsion in an extremely low birth weight infant that occurred during vaginal delivery. Scalp avulsion can cause major bleeding and can be fatal. Bleeding control and circulation maintenance are the most important factors in its management. Suturing the wound effectively controlled bleeding, and the high-humidity environment inside the closed incubator may have contributed to scalp engraftment.

## Data Availability

The data used to support the findings of this case report are included within this article.

## References

[1] Moczygemba C. K., Paramsothy P., Meikle S. (2010). Route of delivery and neonatal birth trauma. *American Journal of Obstetrics and Gynecology*.

[2] Hughes C. A., Harley E. H., Milmoe G., Bala R., Martorella A. (1999). Birth trauma in the head and neck. *Archives of Otolaryngology - Head and Neck Surgery*.

[3] McKee-Garrett T. (2019). Delivery room emergencies due to birth injuries. *Seminars in Fetal and Neonatal Medicine*.

[4] Martin R. J., Fanaroff A. A., Walsh-Sukys M. C. (2010). *Fanaroff and Martin’s Neonatal-Perinatal Medicine: Diseases of the Fetus and Infant*.

[5] Matsubara S., Usui R., Koike Y., Gomi A. (2013). Birth injury after cesarean section at 24 weeks of gestation: a large scalp laceration. *Archives of Gynecology and Obstetrics*.

[6] Durham H., Sekula-Perlman A., Callery R. T. (1998). Iatrogenic brain injury during emergency cesarean section. *Acta Obstetricia et Gynecologica Scandinavica*.

[7] Kalra G. S., Goil P., Chakotiya P. S. (2013). Microsurgical reconstruction of major scalp defects following scalp avulsion. *Indian Journal of Plastic Surgery*.

[8] Hung Y. C., Huang J. J., Hsu C. C. (2009). Emergency management of total scalp avulsion. *Emergency Medicine Journal*.

[9] Cunningham F. G., Williams J. W. (2014). *Williams Obstetrics*.

[10] Leedy J. E., Janis J. E., Rohrich R. J. (2005). Reconstruction of acquired scalp defects: an algorithmic approach. *Plastic and Reconstructive Surgery*.

[11] Nguyen H. H. (2012). The microsurgical replantation of seven complete scalp avulsions: is one artery sufficient?. *Journal of Plastic, Reconstructive & Aesthetic Surgery*.

[12] Ashton M. W. (2021). Tips on raising reliable local perforator flaps. *Plastic and Reconstructive Surgery – Global Open*.

